# Books and Pamphlets Received

**Published:** 1888-07

**Authors:** 


### BOOKS AND PAMPHLETS RECEIVED.

*The Homiletic Review* for June is fully up to the average in point of ability and homiletic value. Dr. Princeton has a grand article on "Historical Studies," as of value to the preacher. Bishop A. Cleveland Cox discusses "Apologetics in the Pulpit," deprecating their frequent and indiscriminate use. Prof. Bloombergh completes his very scholarly presentation of the Religious and Moral Views of Horace. Prof. Schodde gives a very interesting sketch of the Theology of the German Universities "Were all Mankind from One Pair?" is discussed by Dr. C. S. Robinson in a light somewhat new and startling to orthodox readers. Published by FUNK & WAGNALLS, 18 and 20 Astor Place, New York. $3.00 per year; 30 cents per single number.

*Modern Methods of Antiseptic Wound Treatment.* Compiled from notes and suggestions from the following eminent surgeons: D. Hayes Agnew,

M.D., LLD.; A. C. Bernays, M.D.; S. W. Gross, M.D , LL.D.; Hunter McGuire, M. D., LL. D.; Thomas G. Morton, M. D.; N. Senn, M. D.; Stephen Smith, M.D.; Lewis A. Stimson, M.D.; J. William White, M.D. Published by Johnson & Johnson, New York.

The aim of the compilers of this little book, as stated in the preface, is to present concise information concerning the details of the application of the aseptic and antiseptic methods in surgery, and therein supply a deficiency in most of the surgical text-books, which, on account of the comparatively recent origin of antisepsis, are lacking in this respect. The book contains a short review of antiseptic progress, a table showing the value of various germicides tested bacteriologically by Dr. John E. Weeks, of New York, and a list of the most used and necessary articles needed in the method, with general directions for their application.

*Annual of the Universal Medical Sciences.* A yearly report of the progress of the general sanitary sciences throughout the world. Edited by Charles E. Sajous, M.D., Lecturer on Laryngology and Rhinology in Jefferson Medical College, Philadelphia, etc., and seventy associate editors, assisted by over two hundred corresponding editors, collaborators and correspondents. Illustrated with chromo-lithographs, engravings and maps. Philadelphia and London. F. A. Davis, Publisher. 1888.

We have in this work a great and important undertaking in which the editor has displayed a prodigious energy. The scheme of undertaking " to collate the progressive features of medical literature at large, and clinical data from countries in which no literature exists, and to present the whole once a year in a continued form " is as brave in its inception as it must prove herculean in its labors. As a guarantee of the editors ability for this great enterprise we have already before us, upon our editorial table, five large octavo volumes for 1888. Aggregating 2,768 pages. The work is ably gotten up and neatly printed in large, plain and beautiful type. The illustrations are many and excellent, and the matter of the work varied, interesting and practical. In the first volume we note that " Diseases of the Brain and Spinal Cord," " Fevers," " Mouth and Stomach," " Intestines and Pancreas," " Parasites," " Rheumatism and Gout," Supra Renal Capsules," " Blood and Spleen," " Tuberculosis and Scrofula," and " Urynalesis " elaborately treated by such men as Seguin, Wilson, Thompson, Johnston, Leidy, Davis, Tyson and Guiteras. And so the several volumes are alike ably discussed by strong writers, and all the subjects illustrated, being brought down to the latest advances. The work should find its way to the library of every progressive medical man.

*The People's Hand-Book on Immersion,* Infant Baptism, Close Communion, and Plan of Salvation, or Justification by Water *vs.* Justification by Faith, with a chapter on The Campbellite, by Rev. Z. A. Parker, of the North Alabama Conference. Author of Tithes and Prohibition Manual. Second edition revised and enlarged. Nashville, Tenn. Publishing House M. E. Church, South. J. D. Barbee, Agent, 1888.

Doctors like to read on other subjects than medicine; hence we are pleased to receive and review works of every kind which are likely to prove instructive and interesting to our readers. The above work presents in very plain and forcible manner the views of the author on the subjects mentioned. Those desiring to inform themselves upon these important topics would do well to order this work, which is ably and concisely written, presenting in a nutshell all that need be known on the matters referred to.

*Questions and Answers on the Essentials of Physiology*, prepared especially for students of medicine. By H. A. Hare, B. Sc., M. D., Demonstrator of Therapeutics and Instructor in Physical Diagnosis in the Medical Department of the University of Penn.; Physician to the Dispensary for the Diseases of Children, etc., etc., with illustrations. Philadelphia: W. B. Saunders. 1888.

This is a very condensed and useful little work of 170 pages on physiology, not intended, as the author states, to supplant any of the text books, but to contain, as its title declares, the essence of those physiological facts with which the average student must be familar.

*The Year Book of Treatment for* 1887. A critical review for practitioners of medicine and surgery. Philadelphia: Lea Brothers & Co. 1888.

The above work is a book of 336 pages and is designed "to present to the practitioner not only a complete account of all the more important advances made in the treatment of disease, but to furnish also a review of the same by competent authorities.

*Manual of Clinical Diagnosis.* By Dr. Otto Seifert, *Privat docent* in Wurzburg, and Dr. Freidrich Muller, *Assistant*, Der 11 Med. Klink in Berlin. Third edition, revised and corrected by Dr. Freidrich Muller, Translated, with the permission of the authors, by William Buckingham Cranfield, A. M., M.D., Berlin, Fellow of the American Academy of Medicine ; Member of the Medical and Chirurgical Faculty of Maryland; Visiting Physician to the Union Protestant Infirmary of Baltimore; Lecturer on Normal Histology and Chief of Clinic for Throat and Chest, University of Maryland, with six illustrations. New York and London: G. P. Putnam's Sons. 1887.

We have here a book of 175 pages ably written and containing an array of most valuable facts in Clinical diagnosis. It is eminently useful and practical, and worthy of the careful perusal and study of every practitioner of medicine. A dose table is found in the latter part of the work. For hypodermic use one half the dose is advised. For the rectum twice the dose by the mouth, and for children the dose is found by adding 12 to the age of the child and dividing by the age; thus: for a child 4 years old the dose would be $\frac{12x24=4}{4}$ or ¼ of the dose for adults.

